# Cyclophosphamide leads to persistent deficits in physical performance and in vivo mitochondria function in a mouse model of chemotherapy late effects

**DOI:** 10.1371/journal.pone.0181086

**Published:** 2017-07-10

**Authors:** Marie-Laure Crouch, Gary Knowels, Rudolph Stuppard, Nolan G. Ericson, Jason H. Bielas, David J. Marcinek, Karen L. Syrjala

**Affiliations:** 1 Clinical Research Division, Fred Hutchinson Cancer Research Center, Seattle, Washington, United States of America; 2 Department of Radiology, School of Medicine, University of Washington, Seattle, Washington, United States of America; 3 Translational Research Program, Public Health Sciences, Fred Hutchinson Cancer Research Center, Seattle, Washington, United States of America; 4 Human Biology Division, Fred Hutchinson Cancer Research Center, Seattle, Washington, United States of America; 5 Department of Pathology, University of Washington, Seattle, Washington, United States of America; 6 Department of Psychiatry and Behavioral Sciences, School of Medicine, University of Washington, Seattle, Washington, United States of America; Roswell Park Cancer Institute, UNITED STATES

## Abstract

Fatigue is the symptom most commonly reported by long-term cancer survivors and is increasingly recognized as related to skeletal muscle dysfunction. Traditional chemotherapeutic agents can cause acute toxicities including cardiac and skeletal myopathies. To investigate the mechanism by which chemotherapy may lead to persistent skeletal muscle dysfunction, mature adult mice were injected with a single cyclophosphamide dose and evaluated for 6 weeks. We found that exposed mice developed a persistent decrease in treadmill running time compared to baseline (25.7±10.6 vs. 49.0±16.8 min, *P* = 0.0012). Further, 6 weeks after drug exposure, in vivo parameters of mitochondrial function remained below baseline including maximum ATP production (482.1 ± 48.6 vs. 696.2 ± 76.6, *P* = 0.029) and phosphocreatine to ATP ratio (3.243 ± 0.1 vs. 3.878 ± 0.1, *P* = 0.004). Immunoblotting of homogenized muscles from treated animals demonstrated a transient increase in HNE adducts 1 week after exposure that resolved by 6 weeks. However, there was no evidence of an oxidative stress response as measured by quantitation of SOD1, SOD2, and catalase protein levels. Examination of mtDNA demonstrated that the mutation frequency remained comparable between control and treated groups. Interestingly, there was evidence of a transient increase in NF-ĸB p65 protein 1 day after drug exposure as compared to saline controls (0.091±0.017 vs. 0.053±0.022, *P* = 0.033). These data suggest that continued impairment in muscle and mitochondria function in cyclophosphamide-treated animals is not linked to persistent oxidative stress and that alternative mechanisms need to be considered.

## Introduction

Traditional cytotoxic agents remain the mainstay of cancer treatment, despite recent progress in cancer therapeutics with the advent of targeted therapies and immunotherapies. Long-term and late toxicities as a result of anti-cancer treatment are common and may include numerous muscle-related complications such as skeletal muscle weakness and fatigue, cardiomyopathies and other forms of heart disease. Across ages, diagnoses and treatments, the dominant symptom in long-term cancer survivors is fatigue, with increasing recognition that this persisting fatigue may be related to sarcopenia or muscle wasting [1–5].

Acquired skeletal muscle weakness and atrophy after cancer and its treatment is associated with increased morbidity and mortality in cancer survivors [6–9]. A number of studies have shown that compared to non-cancer controls, breast cancer survivors have higher rates of appendicular muscle mass loss which correlates with higher levels of fasting insulin, decreased bone density, decreased physical function, and increased risk of early frailty and all-cause mortality [10–18]. Similar findings are apparent for survivors of prostate cancer who are on androgen deprivation therapy, recipients of hematopoietic stem cell transplant (HCT), and survivors of childhood cancers [19–24].

There is a pressing need to determine the mechanisms for these skeletal and cardiac muscle deficits in cancer survivors to permit targeted interventions that will improve physical function, reduce the development of sarcopenia, improve energetics, and decrease vulnerability to prevalent chronic health conditions that may contribute to accelerated aging. Research into the mechanisms and methods to prevent or reverse chemotherapy-related sarcopenia are limited by the difficulty in examining muscle tissue in humans and the need for evaluation over years. Although published research has demonstrated short term energetics deficits in rodent models [25–31], minimal research has modelled chemotherapy regimens related to long-term skeletal muscle dysfunction [32]. Reactive oxygen species (ROS), inflammatory processes, endogenous glucocorticoid production as well as altered cellular metabolism [31–33] have all been implicated in chemotherapy induced skeletal muscle dysfunction. Despite this, little is known of the processes responsible for late skeletal muscle dysfunction and therefore methods for targeting treatment or prevention remain uncertain.

Cyclophosphamide (Cy), either alone or in combination with other chemotherapy drugs, is widely used for the treatment of breast cancer, sarcomas, leukemia, lymphoma, and in HCT, and has demonstrated in vivo and in vitro acute skeletal muscle effects including pain, stiffness, and weakness [22, 34–42]. End-products of Cy metabolism in the intracellular environment include phosphoramide mustard thought to be the main active molecule responsible for DNA cross-linking and damage leading to apoptosis, and the reactive aldehyde acrolein [43]. In addition to DNA strand break, acute exposure to acrolein can disrupt normal mitochondrial electron transport chain function, generate ROS via lipid and protein damage, lead to accumulation of intracellular calcium, myosin heavy chain breakdown, and ultimately atrophy and death of skeletal myocytes [44–50].

This study represents an initial step toward developing a murine model of cancer treatment-related skeletal muscle late effects suitable for examining methods and interventions aimed at preventing and/or reversing such impairment. We administered a single Cy dose to adult mice and performed follow-up assessments for 6 weeks to test the hypothesis that Cy exposure results in an initial and sustained oxidative stress, mitochondrial dysfunction, atrophy and persistent impairment of skeletal muscle function.

## Materials and methods

### Ethical approval and animals

The study was approved by the Institutional Animal Care and Use Committee of the University of Washington (Protocol No. 4130). Four month old female C57BL/6J mice were purchased from Jackson Laboratory. All mice were exposed to a 12-hour light/dark cycle in a fixed-temperature environment with free access to water and standard mouse chow until immediately prior to experimentation. Mouse body temperatures were maintained at 36° ± 1°C throughout in vivo and in situ experiments. Animals received either a single 300 mg/kg dose of Cy (Sigma) or an equivalent bolus of normal saline solution intravenously through the tail (IV). Following administration of Cy or saline, mice were monitored daily for a week for evidence of distress (hunched posture, weight loss >20%, inactivity). For anesthesia during in vivo magnetic resonance spectroscopy and muscle dissection, mice were injected intraperitoneally (IP) with 0.2–0.6mg/g body weight of Avertin tribromoethanol (Sigma). Sedation was confirmed by changes in respiration and lack of reflex upon toe pinching. If needed, a supplemental dose (0.05–0.15 mg/g body weight) was administered subcutaneously during the procedure. For muscle dissection, mice were injected with Avertin as described above and euthanasia was performed immediately following muscle collection by injecting animals with 2.4 mg/g of Avertin IP.

### Treadmill test

Studies were carried out between 6 pm and 8 pm to observe mice during their more active dark cycle. Mice first underwent two days of acclimation during which they were allowed to explore motionless treadmill lanes for 1 min, become familiar with the motivational shocking grid for 1 minute, and walk at a rate of 20 m/min at a 0° incline for 2 min. On the third day, mice were placed on the stationary treadmill at a fixed 10° incline. The treadmill speed was accelerated to a speed of 30m/min over 5 min. and held at this speed until the mice were no longer able to maintain their position on the treadmill. Time to failure was recorded manually and failure was determined when mice were unable to maintain position on the treadmill despite electrical shock and light prodding for 10 sec. Following training and baseline running measurement, Cy or saline was administered to mice as described in previous section. Treadmill running using the same procedure as for day three was measured again 6 weeks after drug exposure.

### In vivo metabolic spectroscopy

For in vivo nuclear magnetic resonance/optical spectra (MR/OS) experiments mice were anesthetized by intraperitoneal (IP) injection of 0.01 mg/kg body weight of tribromoethanol (“Avertin”, Sigma) dissolved in tert amyl alcohol. The distal hindlimb was shaved and the mouse was suspended by flexible straps in a custom built combined MR/optics probe for use with a 14T vertical bore spectrometer (Bruker) [51]. The distal hindlimb was centered within a horizontal MR solenoid coil tunable to both ^1^H and ^31^P with fiber optic bundles positioned on either side to simultaneously collect MR and optical spectra from the intact limb distal to the knee. After positioning the mouse, MR signal was optimized by shimming the ^1^H of tissue water and optical signal was optimized by adjusting acquisition time. A high signal to noise ^31^P spectrum was acquired under fully relaxed conditions (32 transients, 4096 complex points, 10 kHz sweep width, 25 sec interpulse delay). Dynamic optical (0.5 sec delay) and MR (45° flip angle, 4 transients, 4096 complex points, 10 kHz sweep width, 1.5 sec interpulse delay) spectra were acquired continuously through periods of rest (2 min), ischemia (11 min), and recovery (7 min). After the first minute of rest mice breathed 100% O_2_ for the remainder of each dynamic experiment.

### In vivo spectroscopy data analysis

^31^P MR spectra were exponentially multiplied, Fourier transformed, and manually phase corrected using Bruker Inova software [51]. Optical spectra were collected using WinSpec software (Princeton Instruments). The resulting MR spectra and raw optical spectra files were analyzed using custom written MATLAB software. Details of the analytical approach for in vivo metabolic spectroscopy have been described in detail previously [51, 52]. Resting inorganic phosphate (Pi) and phosphocreatine (PCr) to ATP ratio, P_i_ /ATP and PCr/ATP, respectively were determined from the relative peak integrals from fully relaxed ^31^P MR spectra and used to calculate resting metabolite levels (S1 Table). Three consecutive dynamic spectra were summed to improve signal-to-noise ratio and then the Fit-to-Standard algorithm [53] was used to determine PCr and P_i_ peak magnitudes throughout dynamic acquisition. The ATP concentration from HPLC analysis of mixed muscle was used as an internal reference to calculate absolute PCr and P_i_ concentrations at each timepoint to calculate fluxes. pH was determined using the chemical shift between P_i_ and PCr peaks, and ADP was calculated using the known kinetics of the creatine kinase and adenylate kinase reactions, assuming equilibrium conditions and a Mg^2+^ concentration of 0.6 mM [54–56].

Optical spectra were analyzed using a partial-least squares routine to determine the O_2_ saturations of hemoglobin (Hb) and myoglobin (Mb) throughout dynamic spectral acquisition [52]. Second derivatives of optical spectra were used to minimize the influence of tissue scattering [57]. The concentrations and known O_2_ binding kinetics of Hb and Mb were then used to calculate net O_2_ flux in the closed system of the ischemic hindlimb.

The resting rates of mitochondrial ATP production (ATPase) and O_2_ consumption were calculated during ischemia using least-squares linear approximations of the decline in PCr and O_2_, respectively, during the initial phase of ischemia [51, 52]. The maximum rate of oxidative phosphorylation (ATPmax) was calculated using a least-squares monoexponential approximation of PCr recovery during recovery from ischemia [51, 58, 59].

### Tissue preparation

In preparation for ex vivo mitochondrial respiration measurements at 1 day, 1 and 6 weeks post drug-exposure, mice were anesthetized with Avertin. The extensor digitorum longus (EDL) muscle was removed for respiration assay and the other skeletal muscles (gastrocnemius, tibialis anterior, soleus, EDL, quadriceps) of the distal hindlimb were dissected, weighed, and flash-frozen in liquid nitrogen. From the left leg, gastrocnemius, soleus, and tibialis anterior muscles were pooled and pulverized over liquid nitrogen for measurement of muscle ATP and creatine concentrations by HPLC as described previously [51]. Hb and Mb concentrations were quantified from Coomassie stained gels following SDS-PAGE as described [51]. Gastrocnemius from the right leg was pulverized over liquid nitrogen and prepared for western blotting. All muscle samples were stored at -80°C until the day of assay.

### Citrate synthase

Citrate synthase activity in gastrocnemius homogenates from saline and Cy mice was monitored by spectrometric quantitation (412 nm) of 5,5’dithiobis-2-nitrobenzoic acid conversion to 2-nitro-5-thiobenzoic acid in the presence of Coenzyme A thiol generated during citrate production (CS0720, Sigma).

### Immunoblot analyses

Select subunits of respiratory complexes I through V (NDUFB8-20 kDa, SDHB-30 kDa, UQCRC2-48 kDa, MTCO1-40 kDa, ATP5A-55 kDa; Abcam #110413) were used to measure mitochondrial content. Samples were not heated to avoid degradation of electron transport system subunits. Evidence of oxidative damage and/or stress was measured using specific antibody (4-hydroxynonenal adducts, HNE12-S, Alpha Diagnostic Inc.; SOD1, ADI SOD101, Enzo Life Science; SOD2, ab16956, Abcam; catalase, #219010, Calbiochem). Actin content does not significantly change with age so it was used to normalize protein load [60]. Protein content was determined by densitometry using Bio-Rad imaging hardware and Quantity One software. Representative blots are shown in S1 Fig.

### Ex vivo mitochondrial respiration

Activity of the mitochondrial respiratory chain was measured by monitoring the rate of oxygen consumption with an oxygen electrode (Oroboros Instruments) in the presence of complex specific substrates. Freshly dissected EDL muscle was weighed and placed in ice-cold isolation buffer (100 mM Ca/EGTA, 5.8 mM NaATP, 6 mM MgCl_2_, 20 mM taurine, 15 mM phosphocreatinine, 20 mM imidazole, 0.5 mM DTT, and 50 mM K-MES, pH 7.1). Fascia, fat and connective tissue were removed from muscle fiber bundles under a dissecting microscope, and muscle was carefully separated from surrounding fibers with forceps. Separated fiber bundles held together at either end by the tendons were placed in permeabilization buffer (isolation buffer with 50 μg/mL saponin) and incubated on ice with gentle shaking for 40 min, after which permeabilized EDL was washed twice with isolation buffer and once with respiration buffer (110 mM sucrose, 60 mM potassium lactobionate, 0.5 mM EGTA, 3 mM MgCl_2_, 20 mM, taurine, 10 mM KH_2_PO_4_, 20 mM HEPES, 1 g/L BSA pH 7.1) [61]. Finally, permeabilized EDL muscle fibers in respiration buffer were placed in 2 mL chamber of respirometer and stirred at 25°C. Leak driven (state 4) respiration was determined in the presence of 10 mM glutamate, 5 mM pyruvate, and 2 mM malate and in the absence of adenylates. ADP stimulated respiration (state3, complex I) was measured after the addition of 2.5 mM ADP. The integrity of the outer mitochondria membrane was tested by the addition of 10 μM cytochrome C as described previously [62].Succinate (10 mM) was added to measure maximal flux (state 3, complex I and II). Fully uncoupled respiration was measured in the presence of FCCP (0.1–1 μM).

### Droplet digital (3D) PCR quantification of mitochondria DNA (mtDNA) deletions

MtDNA deletions were measured by droplet digital PCR as described previously [63]. Briefly, total genomic DNA was isolated by phenol/chloroform extraction from previously flash frozen muscle. Ten micrograms were restriction digested with TaqI enzyme and extracted again by phenol/chloroform extraction. Droplet PCR reaction mixture and cycling conditions are described elsewhere [63]. Reaction droplets were made by applying 20 μL of each reaction mixture to a droplet generator DG8 cartridge (Bio-Rad) for use in the QX100 Droplet Generator (Bio-Rad). The thermally cycled droplets were analyzed by flow cytometry in a QX100TM Droplet DigitalTM Reader (Bio-Rad) for fluorescence analysis and quantification of mutation frequencies. The number of target molecules per droplet was calculated automatically by the QuantaSoft software (Bio-Rad) using Poisson statistics as described elsewhere [63].

### Statistical analyses

Unless otherwise noted, values are presented as mean ± SD. For body weight, a general linear model with repeated measures compared the groups over time, with t tests comparing groups within timepoints. Otherwise, significance between groups was determined by unpaired t test. For exercise tolerance, significance within group between baseline and 6 week timepoints was determined by Log-rank (Mantel Cox test).

## Results

### Exercise intolerance in mice treated with Cy

**Fig 1:**
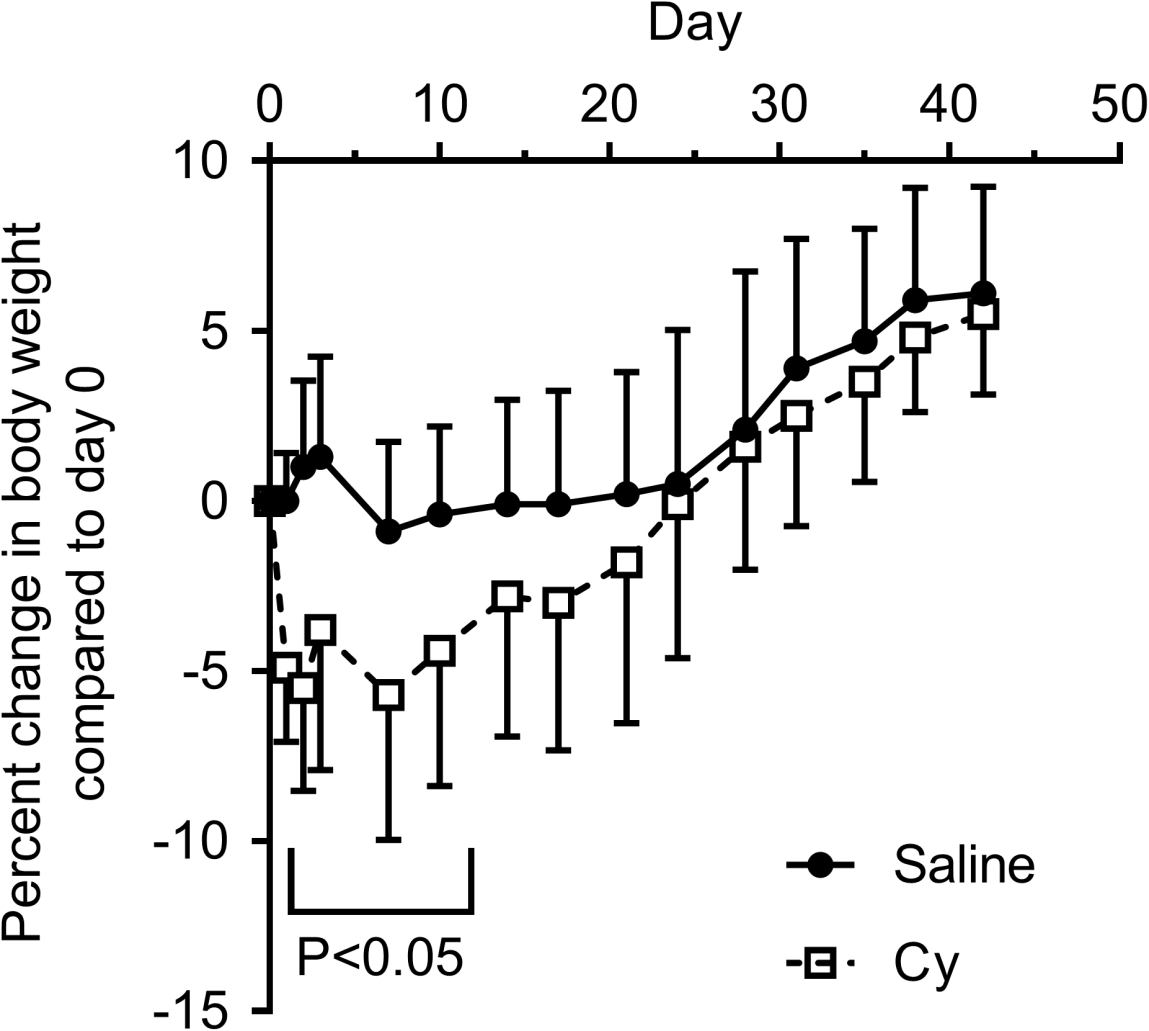
Transient weight loss after exposure to a single Cy dose. Percent body weight change by group (mean and SD) compared to Day 0 (dosing day) for saline and Cy groups, N = 10 per group.

**Fig 2:**
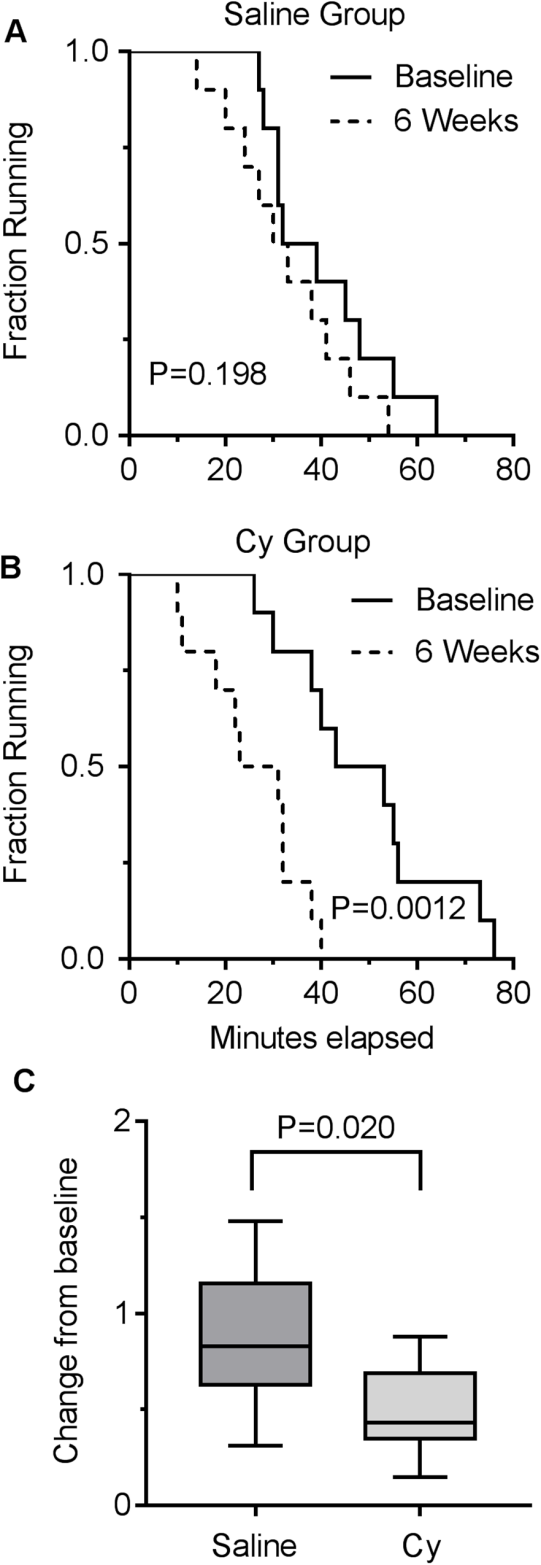
Persistent exercise intolerance in adult mice exposed to a single Cy dose. (A) Saline control group treadmill running capacity in minutes, with no difference between baseline and 6 week timepoints. (B) Cy group treadmill running capacity in minutes, with significant decline in running time between baseline and 6 week timepoints. (C) Fraction change in running capacity between the 2 groups. Data presented as box plot showing min, median, and max data point. N = 10 for each group.

**Fig 3:**
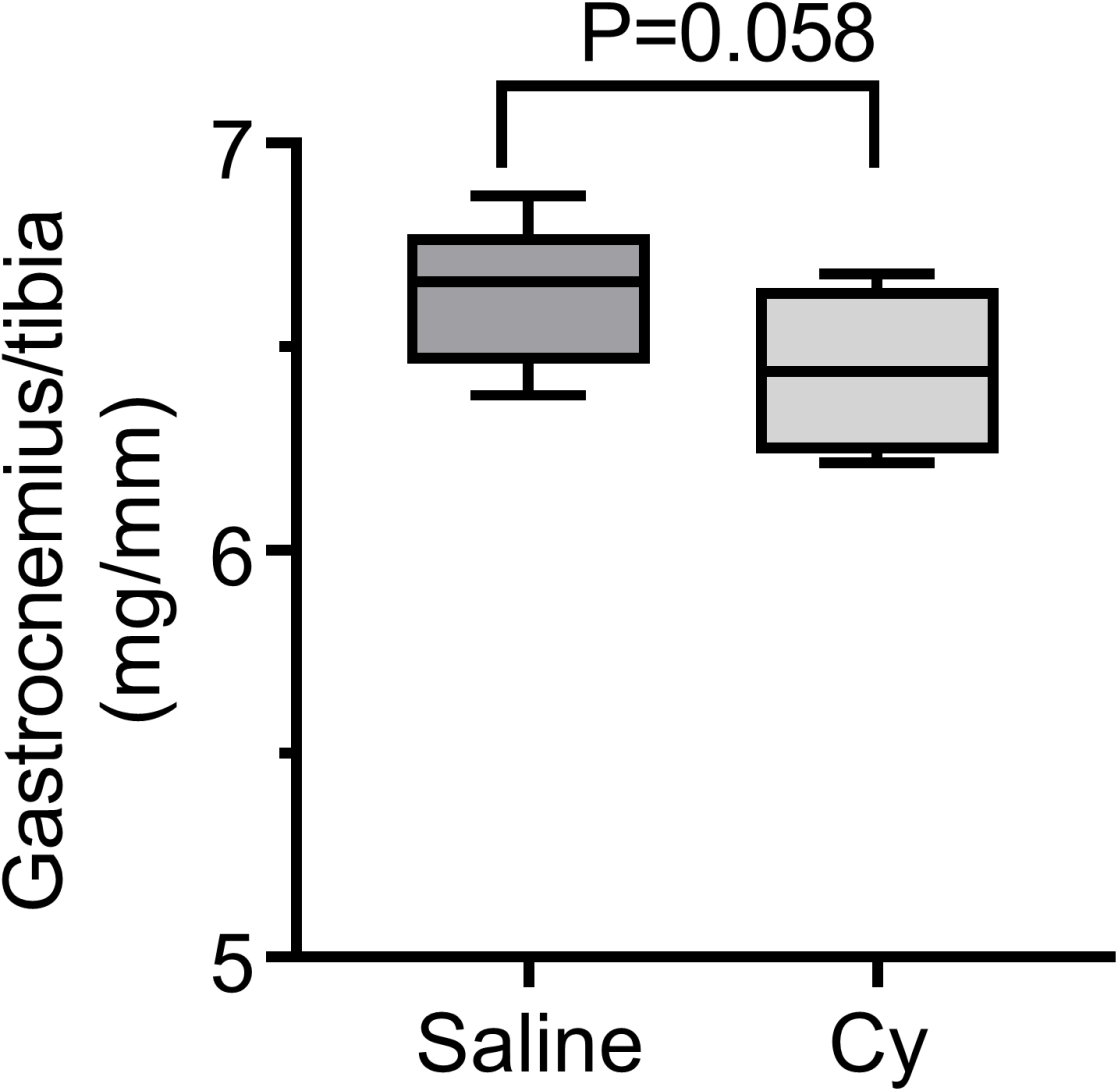
Lower gastrocnemius muscle weight in Cy exposed mice. Gastrocnemius weight relative to tibia length was descriptively smaller in the Cy group than the saline group 6 weeks after treatment, but the difference did not reach P<0.050. Data presented as box plot showing min, median, and max data point. N = 8 for each group.

Mouse body weight after exposure to a single 300 mg/kg Cy dose or saline was monitored throughout the follow up period. Mean body weight of Cy group initially decreased by 5% from baseline one day post-infusion with subsequent gradual recovery to baseline by day 25, with a significant interaction between groups over time (P = 0.002, Fig 1). Change in whole-body exercise capacity was determined by measuring the time to running exhaustion on a treadmill at the start of the experiment (baseline) and 6 weeks after drug exposure. Running performance in the saline control group remained unchanged between the two timepoints (*P* = 0.198, Fig 2A and 2C). In contrast, at 6 weeks the Cy group declined in exercise tolerance (*P* = 0.0012), indicative of a persistent functional deficit (Fig 2B and 2C). Although the decline in exercise tolerance was highly significant for the Cy group, this difference was not as evident in reduced gastrocnemius mass 6 weeks after Cy treatment relative to controls (P = .058; Fig 3). No differences were observed for the tibialis anterior, soleus, or EDL muscle weights normalized to tibia length (all P>0.4).

### In vivo mitochondrial deficits persist in Cy treated mice

**Fig 4:**
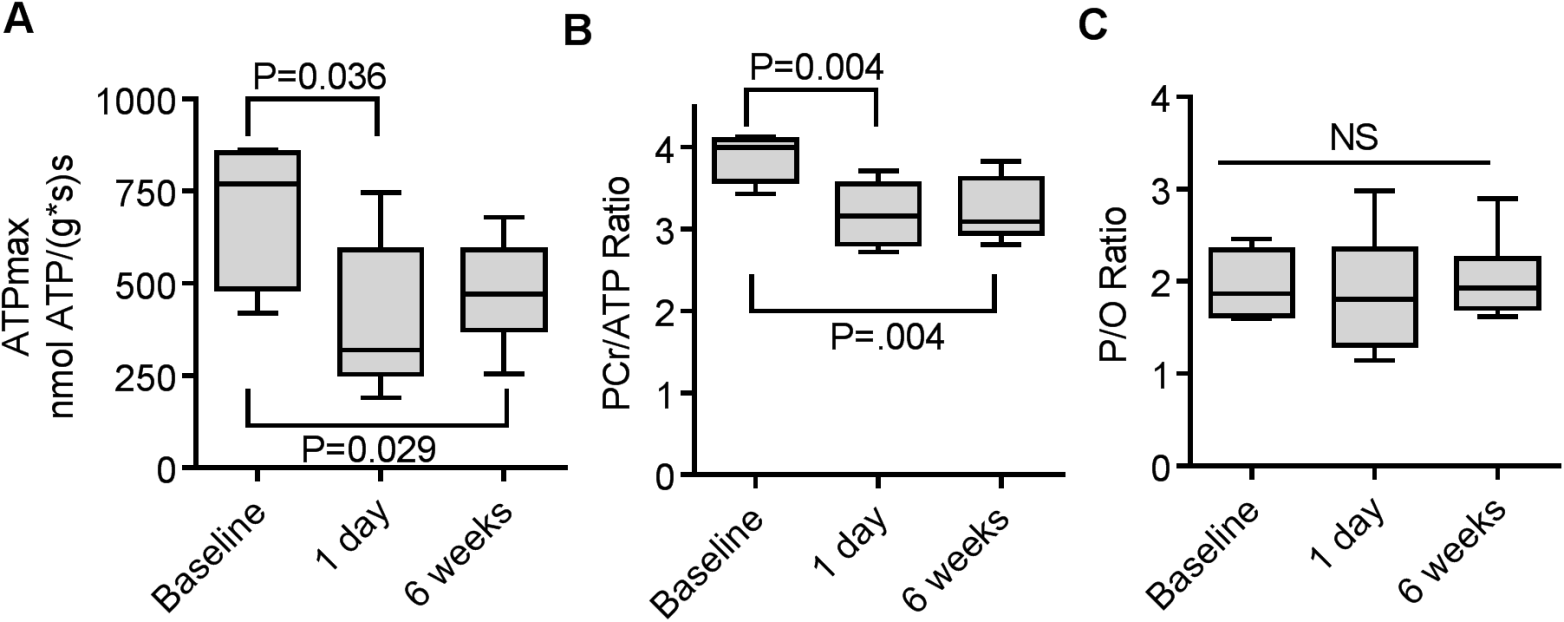
In vivo mitochondria function is impaired after exposure to a single Cy dose. (A) Maximal mitochondrial ATP production (ATPmax) was reduced in the hindlimb skeletal muscles of Cy treated mice compared 1 day and 6 weeks after treatment. In the 1 day dataset one data point that was outside the typical range of values and that failed the outlier test was excluded from the analysis. Excluding this point makes data significantly depressed at P = 0.036. (B) PCr/ATP ratio was calculated from mean PCr and ATP concentrations (S1 Table). Cy treatment led to a reduced PCr/ATP ratio at both timepoints. (C) There was no effect of Cy treatment on coupling of oxidative phosphorylation (P/O). Data presented as box plot showing min, median, and max data point. (N = 5 for baseline and 1 day timepoints, N = 8 for 6 week timepoint) (NS, not significant).

In order to test whether the increased exercise intolerance after Cy treatment was indicative of mitochondrial dysfunction, we measured in vivo mitochondrial energetics using metabolic spectroscopy. Maximum mitochondrial ATP production (ATPmax) significantly declined 1 day after treatment and remained lower than baseline at 6 weeks (Fig 4A) suggesting that Cy treatment induces a persistent metabolic dysfunction. The PCr/ATP ratio, a measure of energy stress in the muscle, also was significantly reduced from baseline at day 1 and week 6 (Fig 4B). Despite the disruption of ATPmax and energy stress we observed no effect of Cy treatment on the coupling of oxidative phosphorylation (P/O, Fig 4C).

### In vitro mitochondrial deficits not apparent in Cy treated mice despite in vivo decline

**Fig 5:**
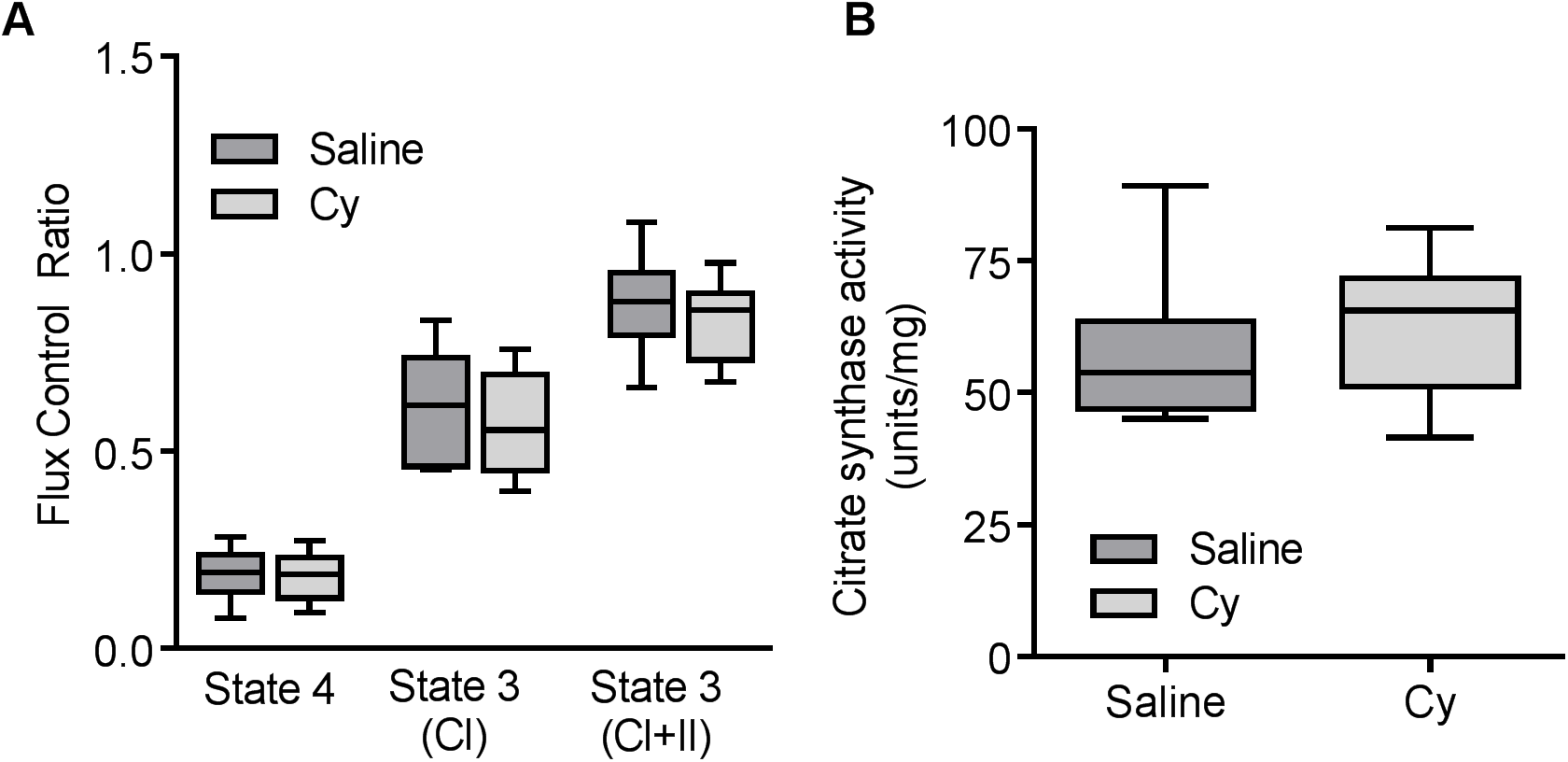
No differences for in vitro mitochondria function 6 weeks after exposure to Cy. (A) Flux control ratios relative to fully uncoupled respiration for state 4, state 3 for complex I (CI) and complexes I+II (CI+II) substrates in permeabilized EDL did not differ between saline and Cy treated mice, (N = 8 for each group). (B) Citrate synthase activity from homogenized gastrocnemius muscle was not different between saline and Cy groups (N = 8 for each group). Data presented as box plot showing min, median, and max data point. P>0.05 for all.

To test whether the in vivo mitochondrial deficits were reflected in ex vivo measures of mitochondrial function we measured mitochondrial respiration in permeabilized muscle fibers of the EDL from saline and Cy exposed mice. To assess mitochondrial quality, data in Fig 5A are expressed as flux control ratios (FCR) to maximum uncoupled respiration using complex I+II substrates. Cy treatment had no effect on the FCR for state 4 or state 3 respiration driven by complex I and complex I+II substrates. We also observed no differences in absolute rates of respiration under these conditions (S2 Fig). Citrate synthase (Fig 5B) activity, as a measure of mitochondrial content, in gastrocnemius muscle homogenates, was also not affected 6 weeks after Cy treatment.

### Transient oxidative stress is evident in skeletal muscles exposed to Cy

**Fig 6:**
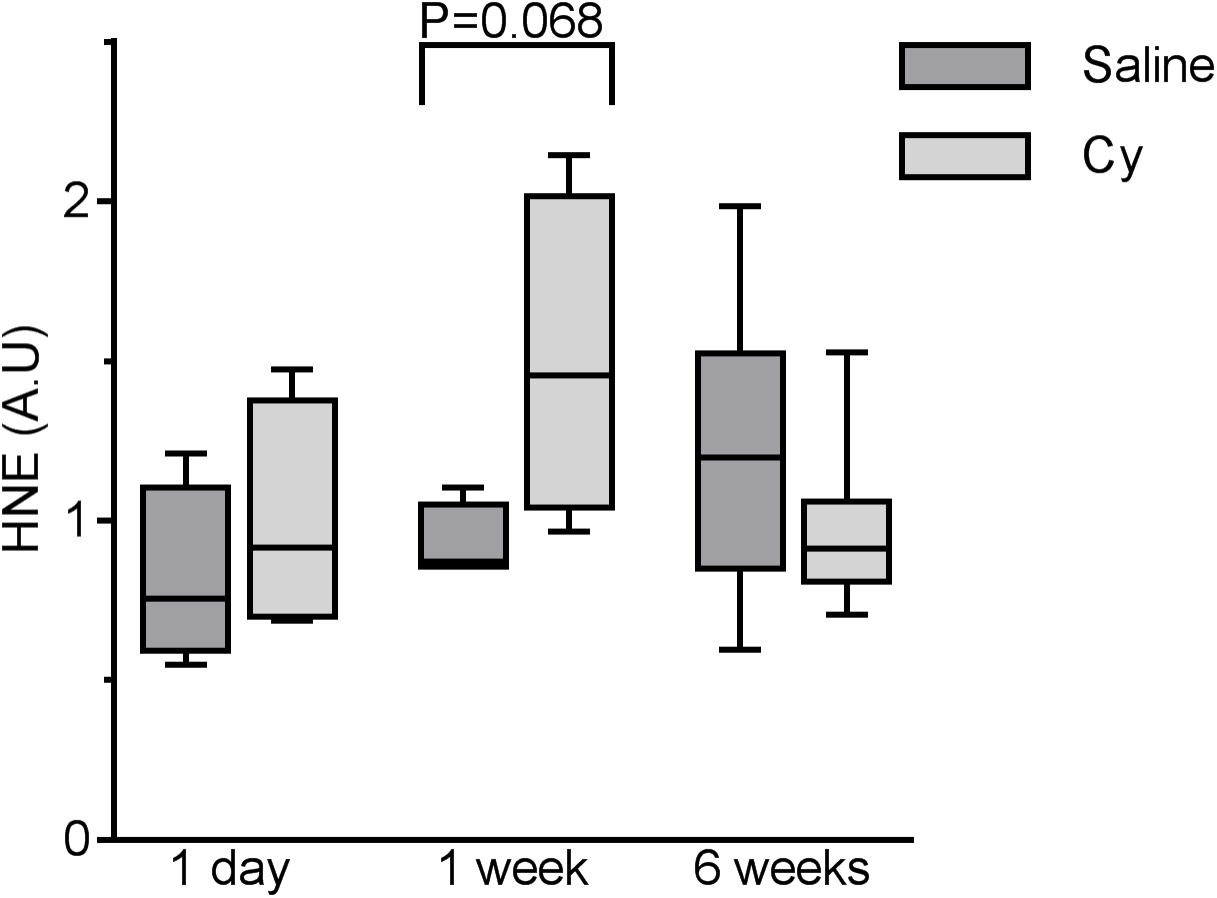
Skeletal muscle of mice exposed to Cy have evidence of mild oxidative damage at 1 week after exposure. SDS-PAGE separation of homogenized EDL muscle followed by immunobloting with specific HNE antiserum. Data presented as box plot showing min, median, and max data point. N = 4 for 1 day and 1 week timepoints and N = 8 for 6 week timepoint.

It has been reported that Cy exerts its cytotoxic effect in part through generation of ROS [64–68]. Hindlimb skeletal muscles were examined for the presence of hydroxynonenal (HNE) protein adducts by quantitative immunoblot detection. Compared to the control group, HNE tended to be higher one week after Cy treatment, with a return to control levels after 6 weeks (Fig 6) suggesting a transient increase in oxidative damage in the skeletal muscle, which appears resolved by 6 weeks. However, levels of Cu, Zn-superoxide dismutase (SOD1) and Mn-SOD (SOD2), as well as catalase remained unchanged throughout compared to untreated controls (S3 Fig). Of note, however, protein levels of the transcription factor NF-ĸB was 58% higher than saline controls one day after drug exposure (*P* = 0.033) and returned to levels similar to controls thereafter (S4 Fig).

### Cy exposure does not result in changes in mtDNA after 6 weeks

In order to assess whether mtDNA mutagenesis was altered due, putatively to the direct effect of Cy induced adducts or elevated oxidative stress, we examined mitochondria DNA mutation frequency by 3D, however no difference in mutation frequency was evident in exposed animals compared to untreated controls (*P* >0.05, S5 Fig).

## Discussion

Understanding of the process by which exposure to Cy results in damage to skeletal muscle and long term impairment in function is limited. Here we demonstrate that a single dose of the alkylating agent Cy in adult mice, compared with saline controlled animals, led to a number of transient (1 day, 1 week) and longer term (6 weeks) skeletal muscle changes. Specifically, 6 weeks after drug administration, we found significant reduction in running capacity. Of note, only the gastrocnemius muscle presented even marginal suggestion of atrophy. Analyses of in vivo mitochondria function showed bio-energetic deficits consistent with impaired mitochondrial function at 1 day and 6 weeks after Cy treatment. The rapid decline in in vivo ATPmax after 1 day may be due to direct inhibition of the mitochondrial electron transport system by the Cy metabolite acrolein [48, 49]. However, the sustained inhibition of ATPmax in the Cy group is likely due to more persistent mechanisms such as disruption of mitochondrial structure resulting from the early toxicity. The absence of an observed effect on respiratory function and mitochondrial content ex vivo despite a decline in in vivo energetics suggests that either 1) the interaction between the cell environment and the mitochondria plays an important role in this Cy-induced dysfunction as we have observed under conditions of oxidative stress and aging skeletal muscle [62, 69] or 2) that in vitro assays performed on permeabilized fibers under saturating substrate concentrations mask more subtle effects on mitochondrial function.

Although NF-ĸB p65 levels were slightly higher in the Cy group at 1 day, along with a marginal but non-significant elevation in HNE at 1 week, we observed no consistent evidence of differences in oxidative stress between the groups. Finally, mtDNA mutation frequency remained comparable to saline control animals suggesting that mtDNA mutations were not a driving factor in the persistent mitochondria dysfunction during the 6-week follow-up.

While Cy is widely used either alone or in combination for the treatment of various cancers, rodent models for Cy toxicities have largely focused on acute effects including cardiovascular [70–74], bladder [75, 76], kidney [77], liver [78, 79], endocrine [80] dysfunctions, changes in taste [81], fatigue and inflammation [82]. Evidence is emerging from our own studies and others that mitochondrial dysfunction as well as oxidative stress, endogenous glucocorticoids increases, inflammation, and metabolic disruption may all contribute to muscle damage after exposure to Cy and other chemotherapeutic drugs [25–28, 30, 32, 33].

Although, our study detected no significant differences in respiratory protein content between control and Cy exposed animals, our examination of in vivo mitochondria bioenergetics in mice after Cy exposure did confirm altered activity as measured by a decrease in maximum ATP production after 1 day and 6 weeks. It is unclear at this time whether the HNE adducts detected at 1 week were the result or a cause of altered mitochondria activity. A single dose of Cy and cisplatin in rats also resulted in a decrease in several parameters of mitochondria health including oxidative phosphorylation, respiratory control ratio (RCR; an index of membrane integrity), and P/O ratio, as well as increased lipid peroxide in liver mitochondria and kidneys after 24 h [83]. In addition to acute effects, chemotherapy drug exposure may lead to long term alterations in mitochondrial function. Lower O_2_ consumption and increased ROS production have been measured in ex vivo respiratory assays of isolated muscle fibers 3 months after doxorubicin and methotrexate exposure in juvenile mice [32]. Similar to our investigation, no changes in proteins of the electron transport system were evident although Parkin levels decreased, suggesting altered mitochondria recycling [32]. Doxorubicin alone can lead to increases in cytosolic antioxidant activity, disrupted mitochondrial energy metabolism, and altered redox balance in cultured C2C12 myotubes as well as mouse skeletal muscle [26, 29].

Also of interest is the potential role of mtDNA mutations as drivers of mitochondria dysfunction and impaired muscle function. Human studies provide evidence both for and against induction of mtDNA mutation due to chemotherapy. Post-mortem examination of mtDNA from cancer patients treated with doxorubicin that had died weeks to months after treatment had no evidence of changes in skeletal muscle mtDNA, while heart mitochondria had detectable levels of mtDNA mutations [84, 85]. A small study of adult survivors of hematologic cancers at least 6 months post-treatment who had received a variety of chemotherapy regimens, did find acquired mtDNA mutation in skeletal muscle tissues although the impact of such mutations is unclear [86]. Mouse models of chemotoxicities have not consistently examined mtDNA in skeletal muscles and those that have, including ours, have so far failed to detect an increase in mtDNA mutation frequency [32, 87].

Strengths of this study include our use of mature mice consistent with the fact that most chemotherapy is administered to mature adults, which is in contrast to the majority of studies where juvenile mice were used. As we aim to develop a translational model of chemotherapy late effects it is important to have animals that are the appropriate age for the chemotherapy regimen tested. Further, as we are interested in late effects, we examined mice for 6 weeks. Future work will continue to follow animals for several months to better determine the trajectory of chemotherapy late effects. Finally, we have made use of a unique tool, MRS-OS spectroscopy, to assess in vivo mitochondria function. As demonstrated by our results, in vitro respiration assays may not reflect more subtle disruption of mitochondria occurring in intact animals. We have found similar results in models of mitochondria aging in mouse skeletal muscle and mild oxidative stress where in vivo deficits are more severe than reflected in ex vivo assays of mitochondria function [62, 69]. Our study did have several limitations. For this study, a single dose of one drug was administered whereas chemotherapy regimens typically consist of a multiple doses of a combination of 2 or more drugs. We are now examining combination regimens designed to parallel treatment regimens used for breast cancer, where research indicates long term muscle deficits after treatment [10–18]. This also explains our selection of only female mice in the study. Assessment of physical function was limited to treadmill endurance running which may be impacted not only by skeletal muscle function but also neurophysiological and cardiopulmonary function. In the future, it will be of interest to examine several parameters of physical function as well as monitoring potential changes in cardiac health. The latter is a critical parameter as several chemotherapy drugs, such as doxorubicin, are known to have acute cardiac effects which may impact skeletal muscle through reduced activity levels.

In conclusion, while we observed a persistent physical deficit and mitochondria dysfunction in vivo after exposure to a single Cy dose, our results do not support a role for persistent oxidative damage and mtDNA mutations as drivers of impaired function. In addition, our focus on skeletal muscle in this study does not address the potential role of cardiac dysfunction in persistent exercise intolerance or other potential sources of muscle atrophy such as disuse related to inactivity. Future directions include the evaluation of a multi-drug/multi-dose chemotherapy regimen to more accurately model those administered to cancer patients, as well as extending analyses to include cardiac function and histopathology for evidence of muscle damage. In addition, it will be critical to correlate altered mitochondrial activity with a functional phenotype such as changes in muscle strength, endurance capacity, and fatigue behavior. Furthermore possible mechanisms other than mitochondrial activity for skeletal muscle dysfunction warrant investigation. Finally, while 4-month old mice are relevant to a young adult cancer survivor population, the majority of cancer survivors are older and it will be of interest to examine the effect of a multi-drug regimen in older animals over a longer period of time to adequately reflect the long term survivorship period and aging in human cancer survivors. Our results are consistent with others demonstrating late effects of chemotherapy on skeletal muscle energetics and performance. Further development of this model will be an important tool to assess the mechanisms underlying the muscles pathologies seen after cancer treatment as well as testing preventive and therapeutic interventions.

## Supporting information

S1 Table Metabolites, Hb and Mb concentrations in distal hindlimb muscles from Cy treated mice. (DOCX)

S1 Fig Representative immunoblots for oxidative stress related antibodies and actin control. Cyclophosphamide (Cy) and Saline (S) gastrocnemius muscle protein homogenates were prepared as described in Materials and methods. Samples were loaded onto each gel randomly and are representative of signals detected for each antibody.
(TIF)

S2 Fig No differences in respiration rate after exposure to Cy compared to saline only. State 3 respiration with complex I+II substrates at (A) 1 day, (N = 4 for each group). (B) 1 week (N = 4 for each group). (C) 6 weeks (N = 8 for each group). Data presented as box plot showing min, median, and max data point. *P*>0.05 for all.
(TIF)

S3 Fig Select antioxidant enzymes remain unchanged after exposure to Cy. SDS-PAGE separation of homogenized EDL muscle followed by immunobloting with antibody specific for A) SOD1, B) SOD2, and C) catalase. Luminescent signal for each was normalized to actin signal. Data presented as box plot showing min, median, and max data point. N = 4 (1 day and 1 week), N = 8 (6 weeks). *P*>0.05 for all.
(TIF)

S4 Fig Levels of NF-ĸB p65 increase shortly after Cy exposure but not at 1 or 6 weeks. NF-ĸB p65 signal was normalized to actin. Data presented as box plot showing min, median, and max data point. N = 4 (1 day and 1 week), N = 8 (6 weeks).
(TIF)

S5 Fig mtDNA mutation frequency in EDL muscles is similar between saline and Cy groups 6 weeks after drug administration. Data presented as box plot showing min, median, and max data point. N = 5 per group.
(TIF)
