# Supplementary material for: Cyclophosphamide leads to persistent deficits in physical performance and in vivo mitochondria function in a mouse model of chemotherapy late effects
Source: PLoS One. 2017 Jul 10;12(7):e0181086. doi: 10.1371/journal.pone.0181086 (PMC5507312; doi:10.1371/journal.pone.0181086)
Supplement: S1 Table — (DOCX) [file pone.0181086.s001.docx]

|  | Baseline | 1 day | 1 week | 6 weeks |
| --- | --- | --- | --- | --- |
| ATP (mM) | 9.42 ± 1.09 | 9.46 ± 1.45 | 9.71 ± 0.55 | 9.77 ± 0.52 |
| PCr (mM)* | 36.67 ± 6.02 | 30.46 ± 7.64 | 32.32 ± 4.91 | 31.72 ± 4.55 |
| P_i_ (mM)* | 2.38 ± 0.55 | 1.58 ± 0.88 | 2.20 ± 1.11 | 2.73 ± 1.01 |
| Cr_total_  (mM) | 41.49 ± 4.65 | 42.12 ± 3.76 | 42.96 ± 2.16 | 44.22 ± 6.51 |
| ADP (µM) | 14.06 ± 7.87 | 33.98 ± 18.42 | 32.50 ± 17.62 | 41.17 ± 21.74 |
| pH_rest_* | 7.06 ± 0.07 | 7.11 ± 0.14 | 7.12 ± 0.09 | 7.14 ± 0.019 |
| Mb (nmol g^-1^) | 0.029 ± 0.007 | 0.035 ± 0.008 | 0035 ± 0.006 | NA |
| Hb (nmol g^-1^) | 0.056 ± 0.015 | 0.050 ± 0.011 | 0.054 ± 0.011 | NA |
| Mbsat_rest_ (%) | 83.6 ± 4.8 | 83.1 ± 9.9 | 85.7 ± 8.8 | 91.4 ± 6.9 |

*Indicates value calculated based on NMR spectra. ATP and creatine 9Cr) concentrations were measured by HPLC. ADP concentration was calculated as described in the text. Total Hb and Mb contents quantitated by SDS-PAGE compared to known standards. Mb saturation was determined from optical spectroscopy according to [[88](#_ENREF_88)]. N=5-8.
