# Supplementary figures and images for: Cyclophosphamide leads to persistent deficits in physical performance and in vivo mitochondria function in a mouse model of chemotherapy late effects
Source: PLoS One. 2017 Jul 10;12(7):e0181086. doi: 10.1371/journal.pone.0181086 (PMC5507312; doi:10.1371/journal.pone.0181086)

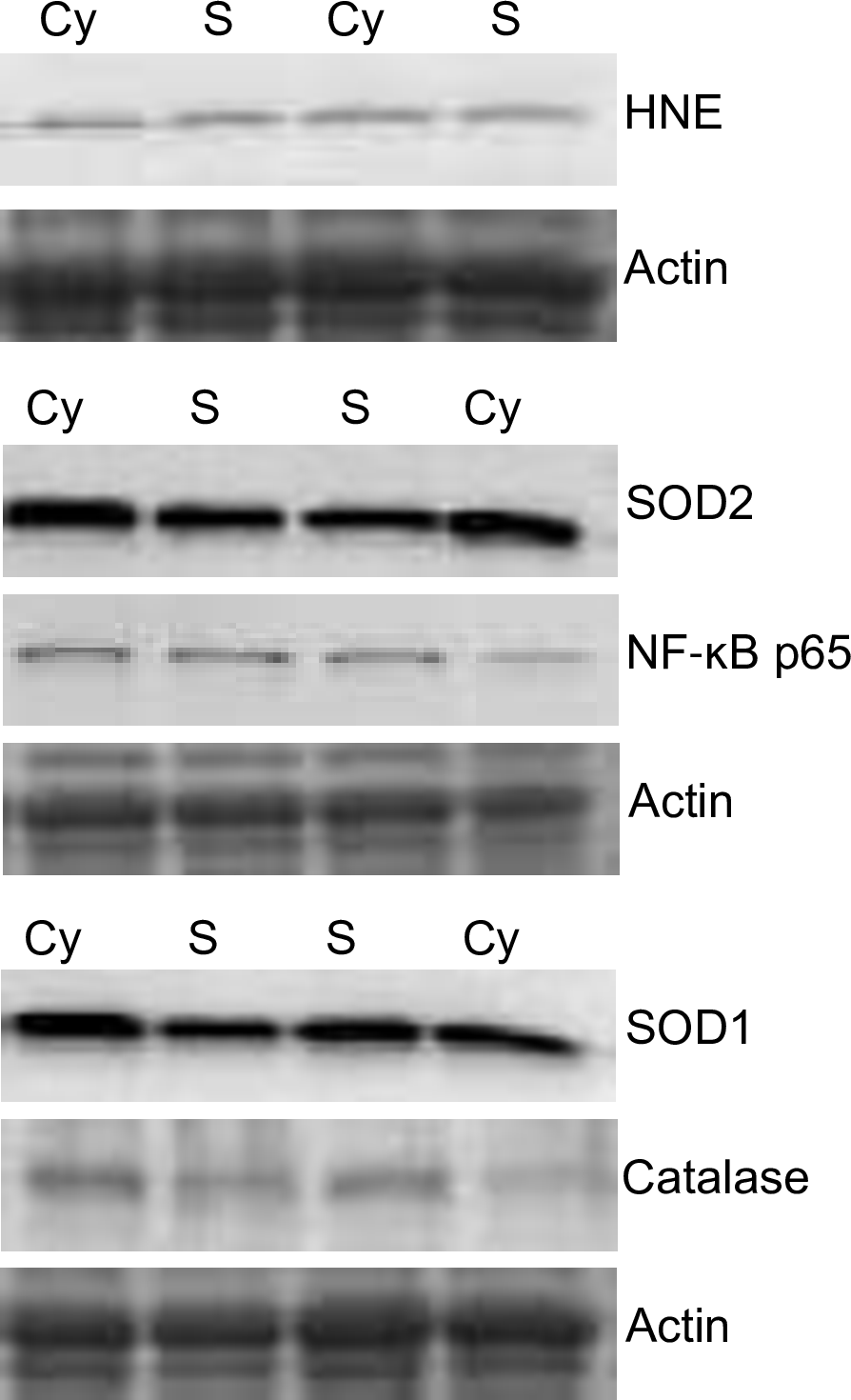

Supplement: S1 Fig — Cyclophosphamide (Cy) and Saline (S) gastrocnemius muscle protein homogenates were prepared as described in Materials and methods. Samples were loaded onto each gel randomly and are representative of signals detected for each antibody. (TIF) [file pone.0181086.s002.tif]

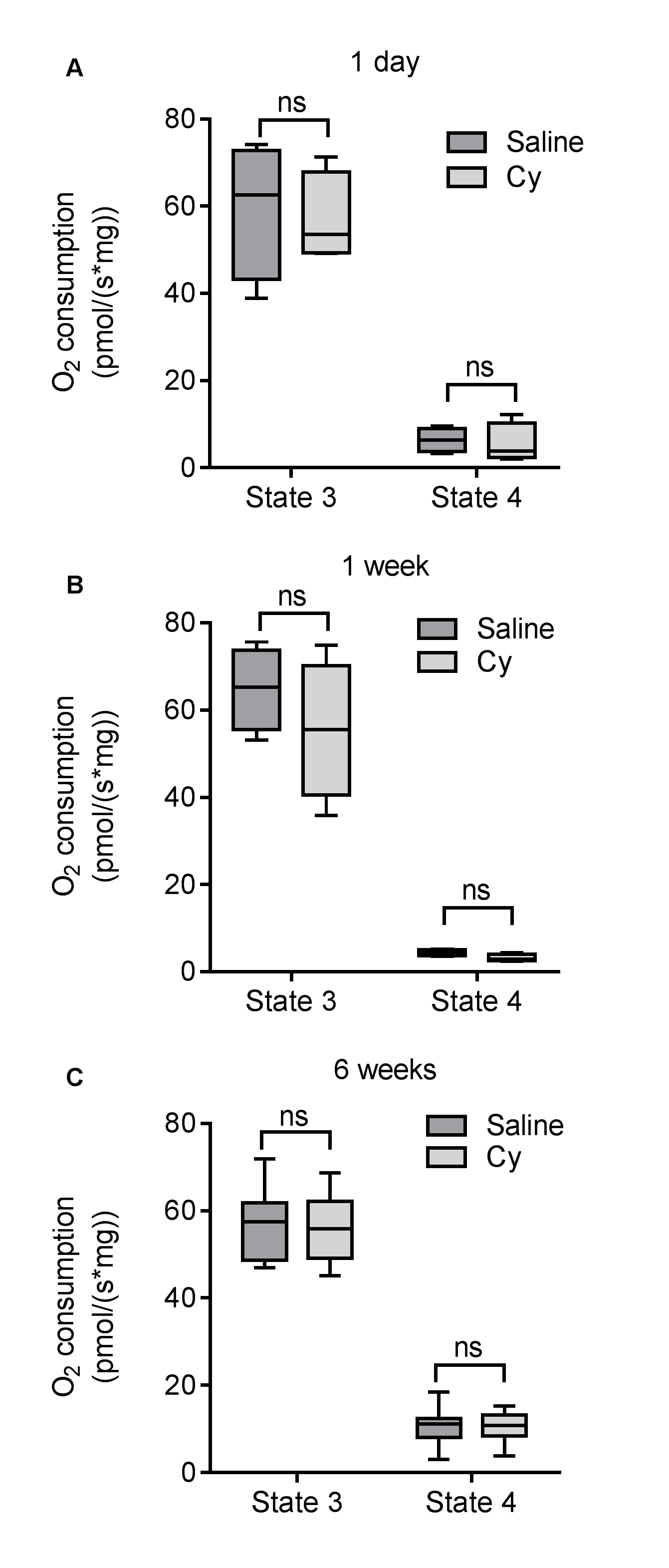

Supplement: S2 Fig — State 3 respiration with complex I+II substrates at (A) 1 day, (N = 4 for each group). (B) 1 week (N = 4 for each group). (C) 6 weeks (N = 8 for each group). Data presented as box plot showing min, median, and max data point. P>0.05 for all. (TIF) [file pone.0181086.s003.tif]

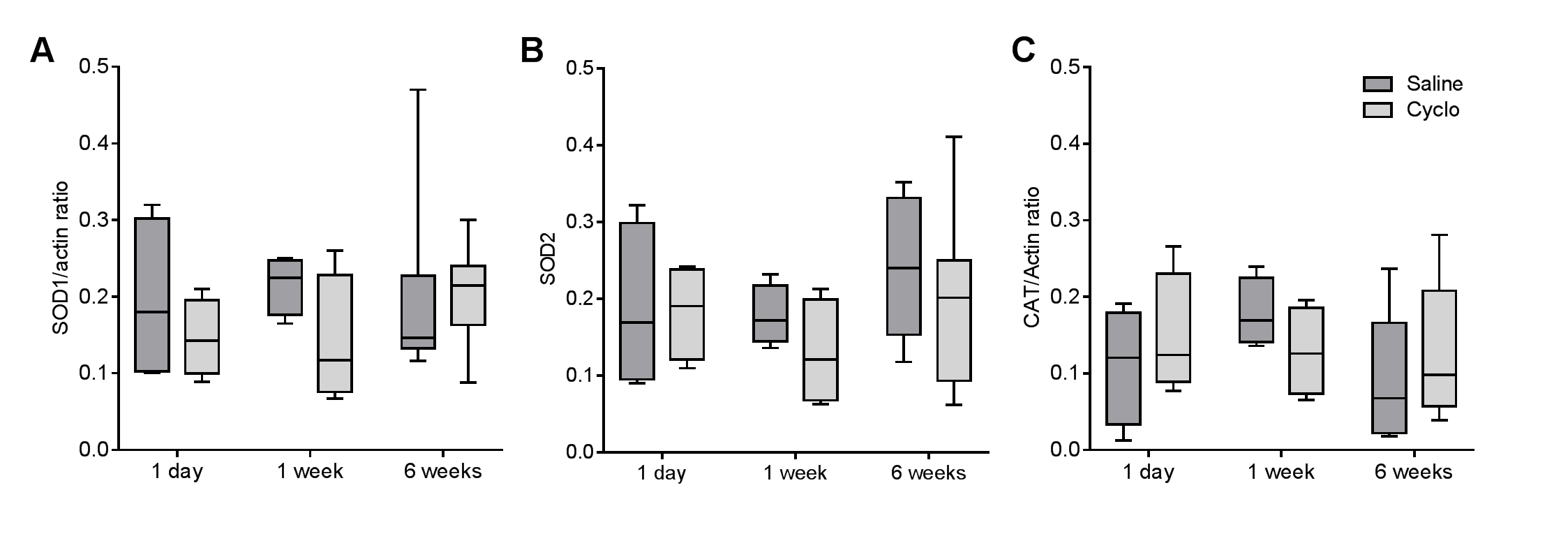

Supplement: S3 Fig — SDS-PAGE separation of homogenized EDL muscle followed by immunobloting with antibody specific for A) SOD1, B) SOD2, and C) catalase. Luminescent signal for each was normalized to actin signal. Data presented as box plot showing min, median, and max data point. N = 4 (1 day and 1 week), N = 8 (6 weeks). P>0.05 for all. (TIF) [file pone.0181086.s004.tif]

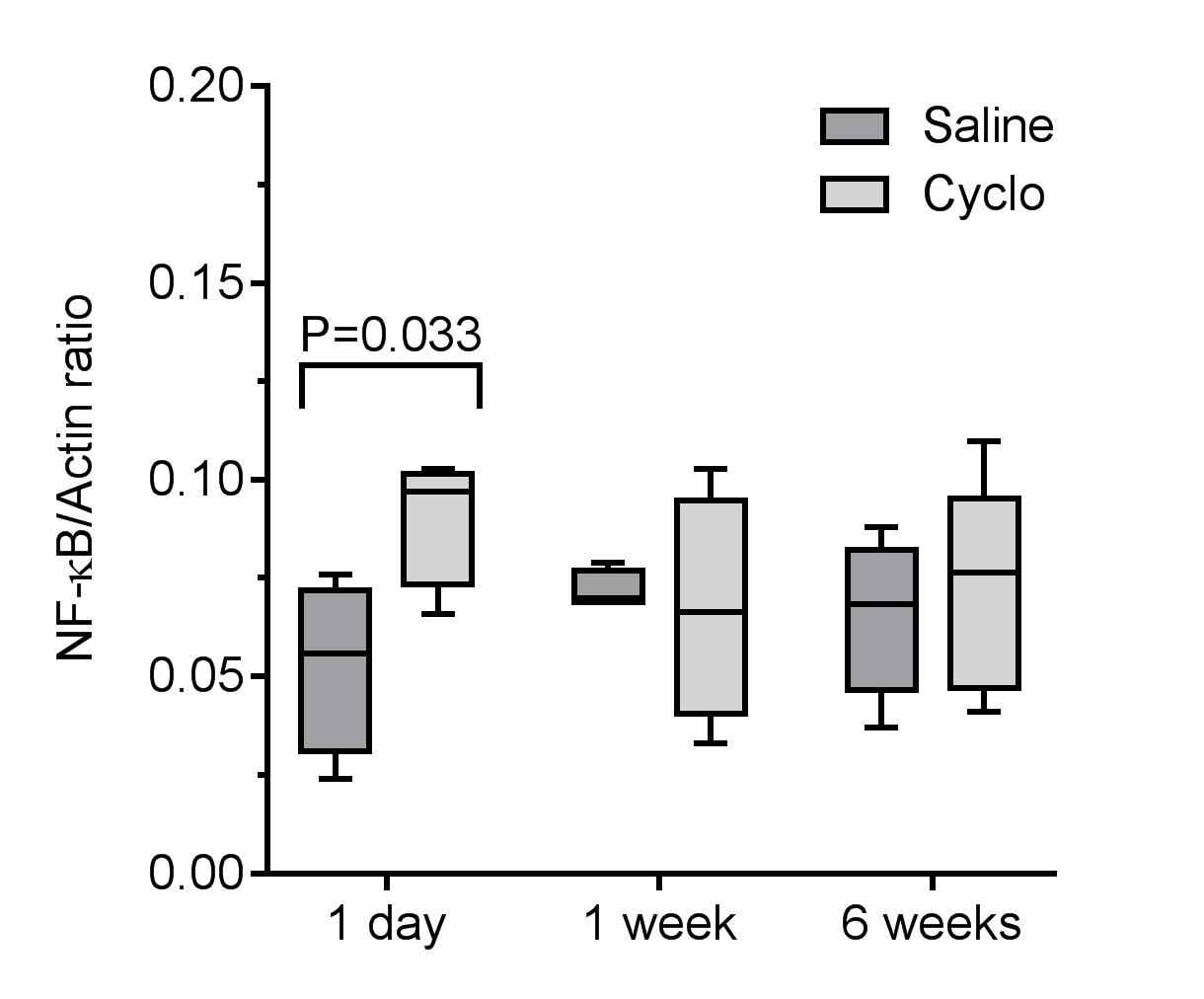

Supplement: S4 Fig — NF-ĸB p65 signal was normalized to actin. Data presented as box plot showing min, median, and max data point. N = 4 (1 day and 1 week), N = 8 (6 weeks). (TIF) [file pone.0181086.s005.tif]

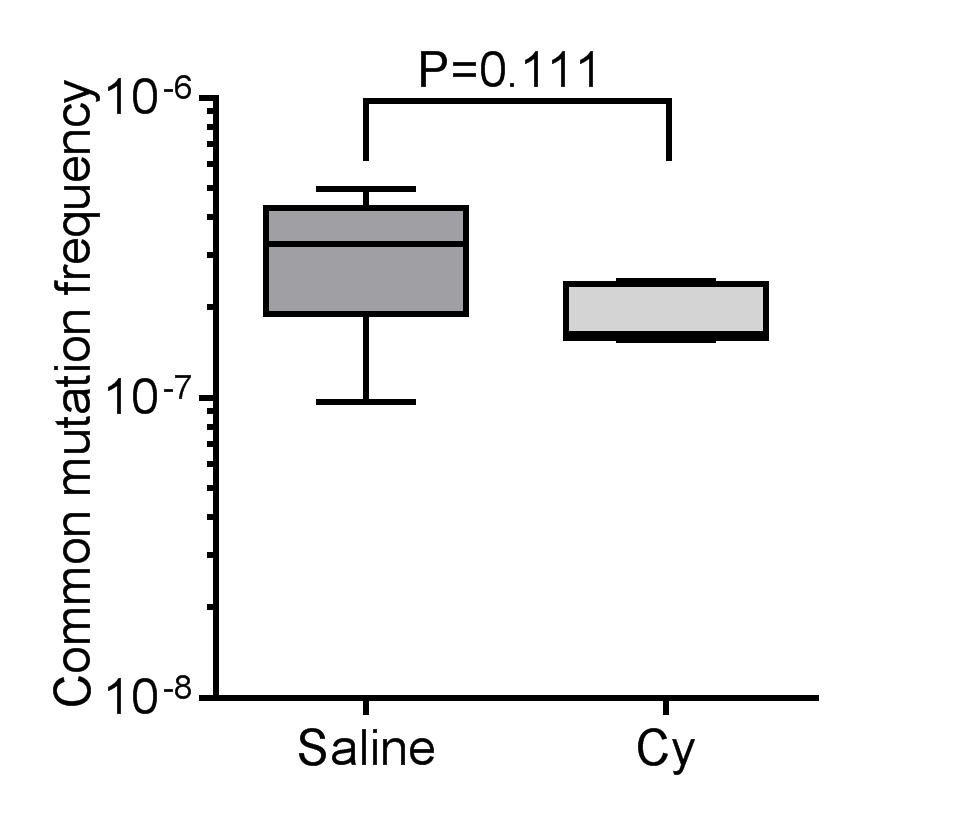

Supplement: S5 Fig — Data presented as box plot showing min, median, and max data point. N = 5 per group. (TIF) [file pone.0181086.s006.tif]
